# A lymphodepleted non‐human primate model for the assessment of acute on‐target and off‐tumor toxicity of human chimeric antigen receptor‐T cells

**DOI:** 10.1002/cti2.1291

**Published:** 2021-06-03

**Authors:** Shigeki Yagyu, Hidemi Mochizuki, Kumiko Yamashima, Hiroshi Kubo, Shoji Saito, Miyuki Tanaka, Kengo Sakamoto, Akihito Shimoi, Yozo Nakazawa

**Affiliations:** ^1^ Department of Pediatrics Graduate School of Medical Science Kyoto Prefectural University of Medicine Kyoto Japan; ^2^ Center for Advanced Research of Gene and Cell Therapy in Shinshu University (CARS) Shinshu University School of Medicine Matsumoto Japan; ^3^ Ina Research Inc. Ina Japan; ^4^ Division of Cancer Immunotherapy Exploratory Oncology Research and Clinical Trial Center National Cancer Center Kashiwa Japan; ^5^ Department of Pediatrics Shinshu University School of Medicine Matsumoto Japan; ^6^ Institute for Biomedical Sciences Interdisciplinary Cluster for Cutting Edge Research Shinshu University Matsumoto Japan

**Keywords:** CAR‐T cells, cell therapy, lymphodepletion, non‐human primate, off‐target toxicity, on‐target toxicity

## Abstract

**Objectives:**

Chimeric antigen receptor (CAR)‐T cell therapy possesses the potential to cause unexpected on‐target toxicities that may be life‐threatening. Non‐human primates (NHPs) share considerable structural homology and expression profiles of most proteins with humans and are therefore utilised as an animal model for non‐clinical safety studies. We have developed a lymphodepleted NHP model by conditioning the animals with immunosuppressive chemotherapy designed to simulate clinical practice conditions, to induce transient mixed chimerism before the administration of human CAR‐T cells redirected to target Ephrin type‐B receptor 4 (EPHB4‐CAR‐T cells) to evaluate the toxicity of these cells.

**Methods:**

We administered 60 mg m^−2^ day^−1^ of fludarabine for 4 days and 30 mg kg^−1^ day^−1^ of cyclophosphamide for 2 days intravenously to cynomolgus macaques for lymphodepletion; then, 3.3 × 10^6^ kg^−1^ of non‐transduced or EPHB4‐CAR‐T cells was infused into the macaques, respectively. All macaques were closely monitored and evaluated for potential toxicity for 7 days.

**Results:**

Lymphodepletion was successfully achieved on day −1 before T‐cell infusion and persisted over 7 days without severe organ toxicities. A single administration of human EPHB4‐CAR‐T cells did not induce overt organ toxicities, although EPHB4‐CAR‐T cells were activated *in vivo* as evidenced by the elevation in copy numbers of the CAR transgene 24 h after infusion.

**Conclusion:**

Although this NHP model is limited for the full evaluation of toxicity of human CAR‐T cells and the conditioning protocol should be further optimised, this lymphodepleted NHP model could be used to assess acute on‐target/off‐tumor toxicities of CAR‐T cells.

## Graphical abstract

We have investigated the on‐target/off‐target toxicity of CAR‐T cells using a lymphodepleted non‐human primate (NHP) model. We followed the most widely accepted non‐myeloablative lymphodepletion protocol in clinical use for CAR‐T therapy to deplete the lymphocyte counts in the macaques, and lymphodepletion allowed CAR‐T cells to transiently expand and become detectable in the peripheral blood. Our lymphodepleted NHP model could provide new insights into the safety assessment of immune effector cell‐based therapy as the investigational new intervention.

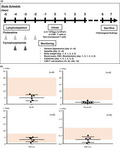

## Introduction

Chimeric antigen receptor (CAR)‐T cell‐mediated cancer therapy has garnered attention as an attractive therapeutic approach. The binding domain of a CAR molecule that is specific for a tumor cell surface antigen comprises a single‐chain fragment variable (scFv) or another binding domain that acts as a specific ligand of the target molecule. Most antigens currently targeted by CAR‐T cells are present on normal tissues with varying levels. In such cases, on‐target/off‐tumor toxicity—defined to be an unintended attack on normal tissues by T cells—is observed. While on‐target/off‐tumor toxicity of CAR‐T cell therapy may be acceptable in certain haematologic malignancies, such as hypogammaglobulinaemia observed after CD19‐CAR‐T cell therapy, it has hindered the development of CAR‐T cells for treating solid tumors and can be life‐threatening in a few cases. For example, severe off‐tumor toxicities have been reported for human epidermal growth factor receptor 2 (HER2)‐specific CAR‐T cells, which possess a HER2‐specific scFv 4D5 in the CAR molecule.^1^ In this report, the patient developed severe respiratory distress within 15 min after T‐cell infusion due to the recognition of negligible HER2 expression on lung epithelial cells by 4D5‐based HER2‐CAR‐T cells.^1^ Another drawback of T‐cell therapy includes the occurrence of off‐target toxicity, which is defined as unexpected promiscuous recognition of unrelated antigens/epitopes derived from normal proteins. Indeed, lethal hyper‐acute off‐target cardiovascular toxicity has been reported in patients with malignant melanoma treated by using melanoma‐associated antigen 3 (MAGE‐A3)‐specific T‐cell receptor (TCR) T cells.^2^, ^3^ Hence, preclinical toxicity evaluation should include not only the expression profile examination of the target antigen but also the actual on‐target binding of CAR‐T cells to the antigen‐expressing normal tissue and off‐target cross‐reactivity.

Since an immunodeficient mouse model would not be suitable for assessing immunological on‐target/off‐target toxicity because of the substantial interspecies divergence between rodents and humans, a non‐human primate (NHP) model has been utilised as an alternative for the safety assessment of T‐cell products.^4^ To evaluate on‐target/off‐target toxicity in an NHP model, the CAR molecule should be introduced into NHP T cells; however, a limitation of this approach would be the differences in the quality of NHP and human CAR‐T cells, as NHP CAR‐T cells cannot completely mimic the properties of the final human CAR‐T cell product used in clinical trials.^5^

Recently, successful induction of transient mixed chimerism has been reported in an NHP model via lymphodepletion.^6^ A combination of cyclophosphamide (CPA) and fludarabine (FDR) could transiently deplete the lymphocytes in NHPs for approximately 2 weeks without causing any severe toxicities to other vital organs and could induce transient mixed chimerism via allogeneic stem cell transplantation.^6^ We hypothesised that if the combination of CPA and FDR could induce sufficient lymphodepletion to facilitate the transient engraftment of xenogeneic T cells, an investigation of both on‐target and off‐target toxicities that might occur immediately after T‐cell infusion would be possible.

We have developed *piggyBac* transposon (PB)‐mediated CAR‐T cells redirected towards the Ephrin type‐B receptor 4 (EPHB4), which is a tumor‐associated antigen expressed not only on various tumors but also on certain normal tissues at low to moderate levels.^7^ We have utilised a natural ligand of EPHB4, Ephrin B2, as an antigen recognition site of the CAR molecule, and these CAR‐T cells effectively recognised and eliminated EPHB4‐positive tumor cells.^7^ EPHB4 and other immunomodulatory molecules are highly conserved between cynomolgus macaques and humans with more than 99% overlap of amino acid sequences (Supplementary figure 1). In this study, we aimed to determine the feasibility of using a lymphodepleted NHP model for the assessment of acute on‐target/off‐tumor toxicity after a single infusion of human peripheral blood mononuclear cell‐(PBMC)‐derived, EPHB4‐specific CAR‐T cells.

## Results

### Lymphodepletion by FDR/CPA successfully induces lymphopaenia in NHPs

**Figure 1:**
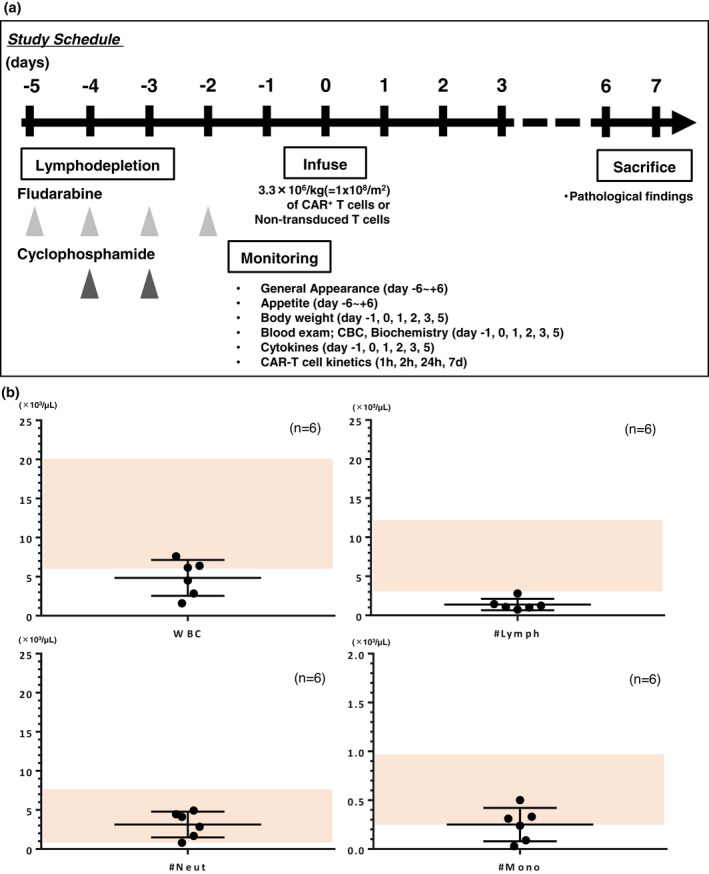
Treatment overview and whole‐blood cell monitoring. **(a)** Treatment overview. **(b)** Whole‐blood cell count on day −1 of T‐cell infusion. The absolute number of total white blood cells, lymphocytes, neutrophils and monocytes of each macaque (*n* = 6) was plotted with mean ± SD. A shaded area represents the normal limits of each parameter.

**Figure 2:**
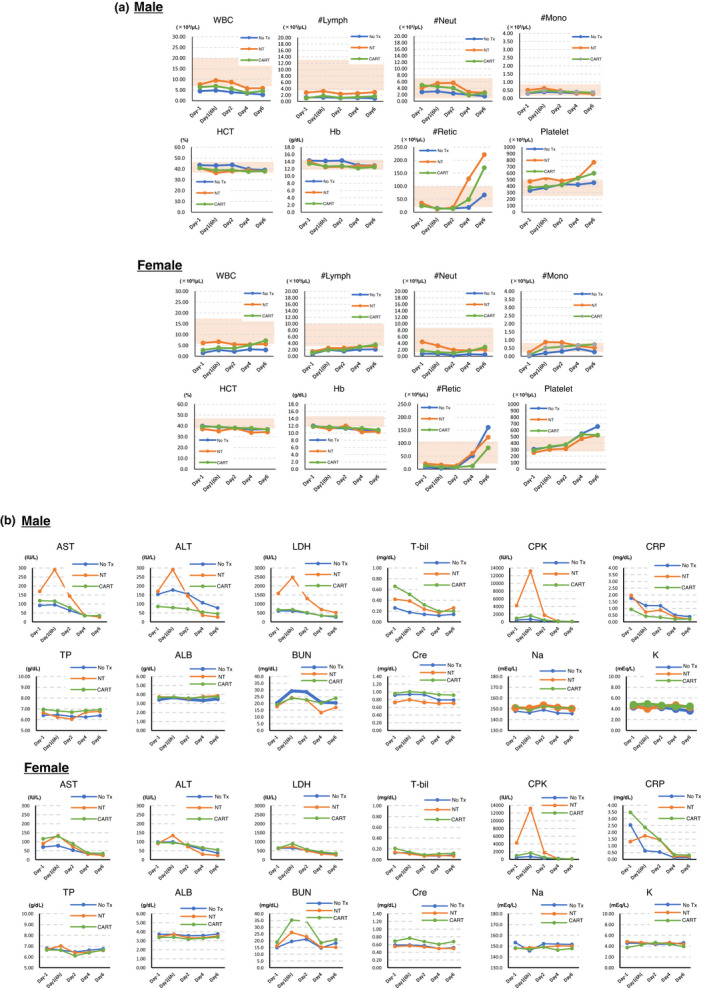
**(a)** Blood cell counts and **(b)** biochemistry in the periphery after performance of conditioning and T‐cell infusion. Each parameter in male and female macaques was measured. A shaded area represents the normal limits of each parameter.

We conducted FDR/CPA conditioning for cynomolgus macaques using the method that had been previously optimised for NHPs (Figure 1a).^6^ Total white blood cell and lymphocyte counts were suppressed below their normal limits in all groups according to the in‐house reference data (Supplementary table 1) in all macaques on day −1 (Figure 1b) and persisted throughout the investigation (Figure 2a) without the occurrence of any overt infections. In contrast, the numbers of neutrophils, monocytes and platelets, as well as haemoglobin levels, were mostly within normal limits,^5^ and the number of reticulocytes increased after day 4 (Figure 2a). These data indicated that the conditioning by FDR/CPA was specific to the reduction in the lymphocyte count and that the suppressive effect on bone marrow function might be transient. Importantly, none of the macaques developed severe conditioning‐related toxicities, such as changes in general appearance, haemorrhagic cystitis and occult bacterial infection. A transient elevation in aspartate transaminase (AST), alanine transaminase (ALT), lactate dehydrogenase (LDH), creatine phosphokinase (CPK) and C‐reactive protein levels was observed prior to T‐cell infusion in all macaques (Figure 2b), which could be attributed to the occurrence of muscular damage because of blood sample collection from macaques in the restrainer or to the adverse effects of the conditioning regimen. These findings indicated that the FDR/CPA conditioning regimen could be used to successfully achieve lymphodepletion without inducing severe toxicities.

### Generation and phenotype of PB‐EPHB4‐CAR‐T cells

**Figure 3:**
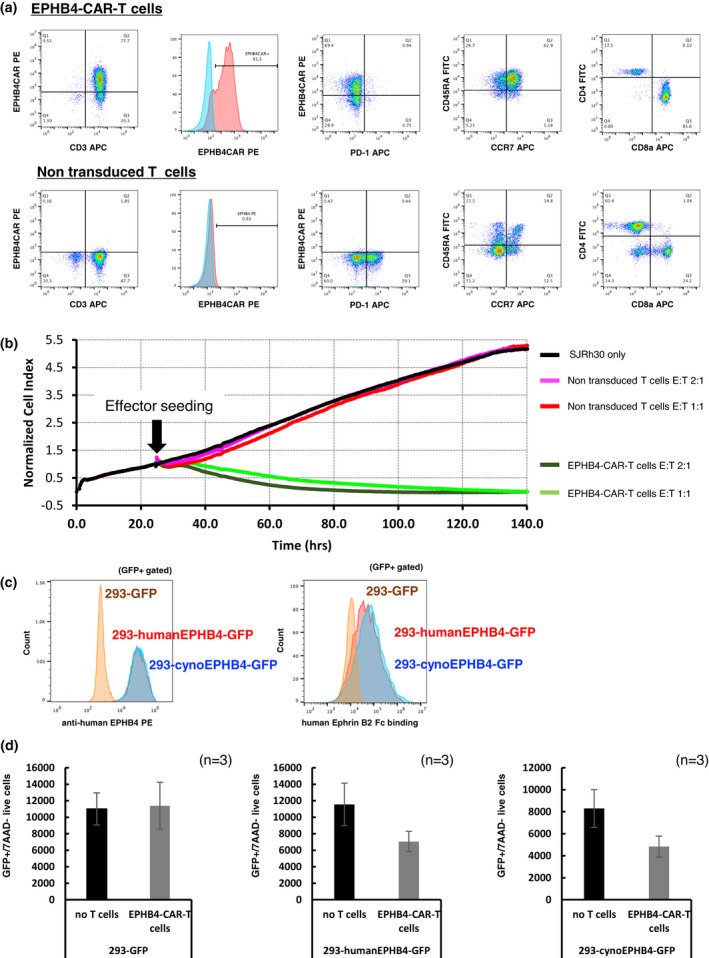
PB‐EPHB4‐CAR‐T cells recognised and killed both human and cynomolgus EPHB4‐expressing cells. **(a)** Phenotype and expression of PD‐1 in EPHB4‐CAR‐T cells and control T cells on day 14 after expansion. **(b)** Killing efficacy of PB‐EPHB4‐CAR‐T cells and control T cells obtained using the xCELLigence^®^ real‐time cell analyser. Rh30 cells were co‐cultured with PB‐EPHB4‐CAR‐T cells or control T cells at E:T ratios of 1:1 and 2:1. The *y*‐axis showed normalised cell index, which represents the relative number of live tumor cells. **(c)** The binding capacity of human Ephrin B2‐Fc chimaera protein to human or cynomolgus EPHB4 molecule. **(d)** Killing efficacy of PB‐EPHB4‐CAR‐T cells on human or cynomolgus EPHB4‐expressing HEK293 cells. The number of live EPHB4‐expressing cells determined in the GFP‐positive/7AAD‐negative fraction was measured using flow cytometry 48 h after the co‐culture. The mean number of live cells in three different experiments is shown. Data were obtained from experiments conducted in triplicate.

We successfully generated EPHB4‐CAR‐T cells with 77.7% CAR positivity (Figure 3a); the total number of cells was 103.5 × 10^6^ after 14 days of expansion. These CAR‐T cells showed skewed CD8 positivity (CD4/CD8 ratio of 0.214) compared to control T cells (CD4/CD8 ratio of 2.495). Notably, EPHB4‐CAR‐T cells predominantly exhibited the CD45RA^+^/CCR7^+^ fraction (62.9%), which was characterised as a stem cell memory phenotype and has been associated with profound and durable antitumor efficacy,^8^ and scarce expression of the exhaustion marker, programmed death 1 (PD‐1) (0.60% ± 0.21%), even after robust cellular expansion. As previously reported, the EPHB4‐CAR‐T cells exhibited strong and durable potency against EPHB4^+^ RH30 cells, as demonstrated via xCELLigence^®^ Real‐Time Cell Analysis (Figure 3b).^7^ These data were consistent with our previous data; therefore, we used these cells for conducting further NHP studies.

We explored the binding and cell‐killing ability of human EPHB4‐CAR‐T cells against cynomolgus EPHB4‐expressing cells. Human recombinant Ephrin B2 possessed a similar binding capacity with human and cynomolgus EPHB4 (Figure 3c), and human EPHB4‐CAR‐T cells could recognise and reduce the number of cynomolgus EPHB4‐expressing cells (Figure 3d). These data indicated the cross‐reactivity of the EPHB4‐binding domain in the CAR molecule with cynomolgus EPHB4.

### Adoptive transfer of human EPHB4‐CAR‐T cells is safe

To evaluate the safety of infusing human EPHB4‐CAR‐T cells into lymphodepleted cynomolgus macaques, we performed a single‐dose infusion of control non‐transduced T cells or human EPHB4‐CAR‐T cells into NT or CAR‐T groups, respectively. As information on the optimised dose of EPHB4‐CAR‐T cells intended for usage in clinical trials was not available, the number of infused cells was considered 3.3 × 10^6^ kg^−1^ (equivalent to approximately 1 × 10^8^ m^−2^) based on the infusion dose used in the ongoing clinical trials targeting sarcomas.^9^, ^10^

We observed no immediate clinical abnormalities related to EPHB4‐CAR‐T cells at the selected dose in 7 days after T‐cell infusion. We limited the observational period to 7 days because of the possible rejection of xenogeneic T cells attributable to immune reconstruction; nevertheless, the observation of symptoms was considered sufficient to deduce preliminary conclusions because most symptoms of life‐threatening acute on‐target/off‐tumor toxicity could have manifested immediately or within several hours to days after T‐cell infusion in the clinical setting.^1^, ^2^ A considerable elevation in CPK levels and a mild elevation in ALT and AST levels were observed on day 1 in all groups, especially in the NT group, which were considered non‐specific T‐cell responses unrelated to CAR (Figure 2b). A transient elevation in blood urea nitrogen (BUN) was observed in a female macaque of the CAR‐T group (Figure 2b). As this tendency was observed in the male macaque of the No Tx group and not observed in the male macaque of the CAR‐T group, it was considered an effect of the conditioning regimen. However, our previous study has revealed that EPHB4 is weakly expressed in renal tissue,^7^ indicating the occurrence of possible renal toxicity. Nevertheless, the elevated BUN levels eventually returned to baseline levels; however, creatinine levels were not affected by the EPHB4‐CAR‐T cell infusion (Figure 2b). Overall, the infusion of EPHB4‐CAR‐T cells did not induce any irreversible acute organ toxicity, although the observation period was limited.

### Transient expansion of PB‐EPHB4‐CAR‐T cells does not induce overt cytokine secretion

**Figure 4:**
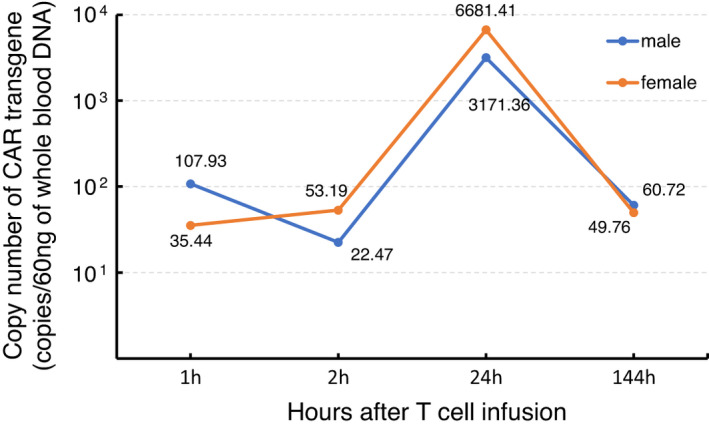
The kinetics of CAR‐T cells after T‐cell infusion. Genomic DNA from the peripheral blood obtained at each time point was subjected to qPCR to detect the presence of the EPHB4‐CAR transgene. Data were obtained from experiments conducted in triplicate.

**Figure 5:**
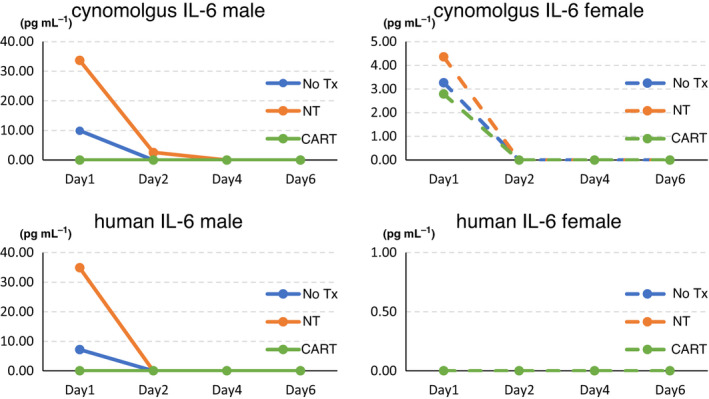
The levels of human and cynomolgus IL‐6 at each time point.

The EPHB4‐CAR transgene was detected in the peripheral blood of the CAR‐T group after infusion, and strikingly, the copy number of the EPHB4‐CAR transgene increased at 24 h after the infusion and then gradually decreased to baseline levels on day 7, indicating the transient expansion of PB‐EPHB4‐CAR‐T cells achieved via antigen stimulation (Figure 4). Transient and mild elevation of IL‐6 levels was observed in a few macaques on day 1; however, the elevation in human and cynomolgus IL‐6 levels was observed in all groups, suggesting that this increase was non‐specific (Figure 5). No pathological elevation in TNF‐α, IFN‐γ, IL‐1β and IL‐2 and levels was observed in this study (data not shown).

### No pathological changes occur in the recipients of human PBMC‐derived EPHB4‐CAR‐T cells

**Figure 6:**
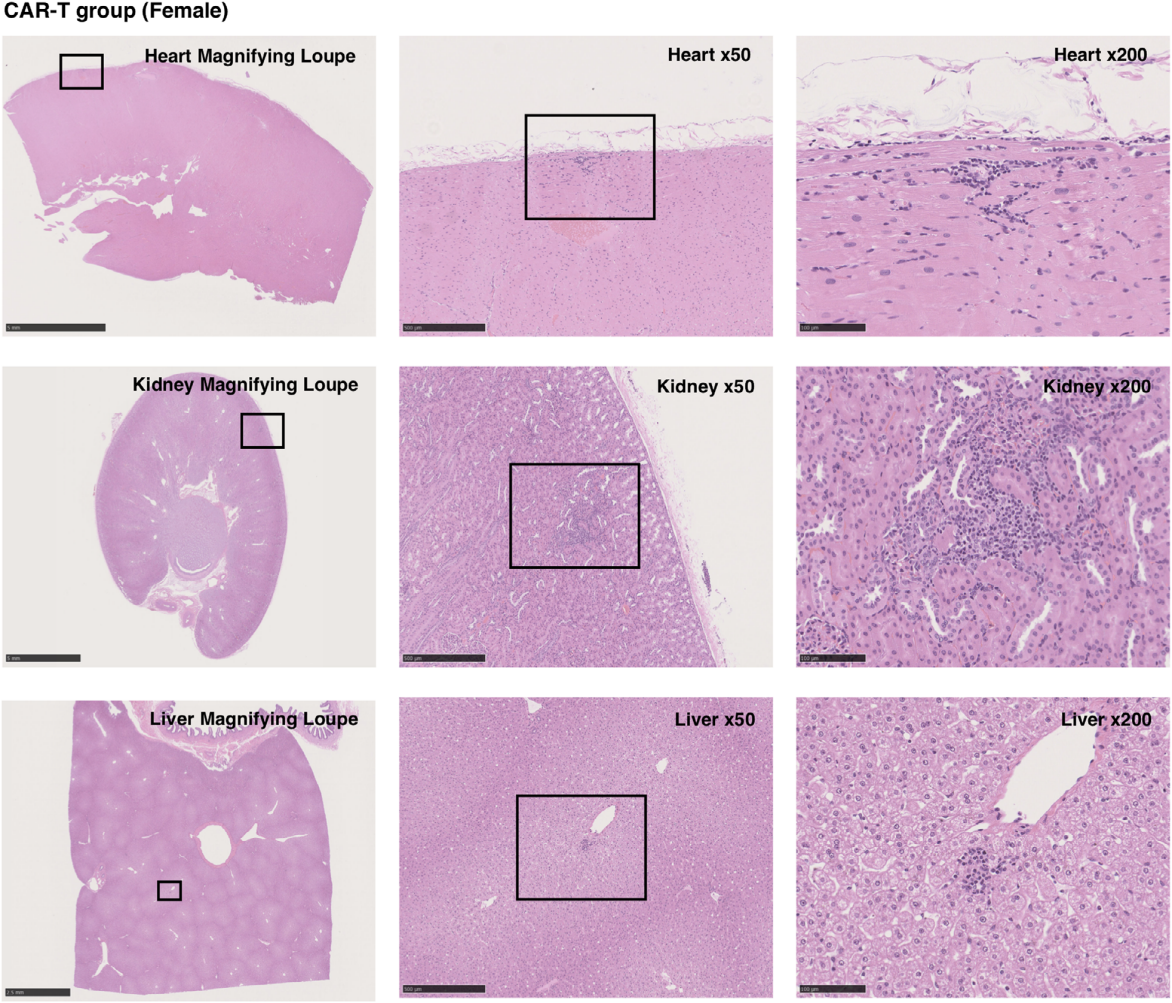
Histological analysis of the CAR‐T group. Representative images of haematoxylin and eosin staining are shown. There were no microscopic changes in the liver, gall bladder, kidney, spleen, heart, brain and thoracic spinal cord because of CAR‐T cell infusion, although markedly few numbers of infiltrating mononuclear cells were detected in the liver, kidney and heart.

The assessment of potential pathological changes in macaque tissues showed no macroscopic changes in the heart, brain, lung, kidney, liver, spleen and spinal cord. Moreover, no microscopic changes related to CAR‐T cell infusion were observed (Figure 6), although the infiltration of cynomolgus mononuclear cells was detected in the liver, kidney and heart, which has been reported in normal cynomolgus macaques,^11^ and the infiltration of cynomolgus mononuclear cells was also detected within the heart of one of the macaques in the NT group (Supplementary figure 2).

## Discussion

In this study, we have successfully developed and established a lymphodepleted NHP model for the assessment of acute organ toxicity of EPHB4‐CAR‐T cells. The conditioning regimen, which consisted of 4 days of FDR and 2 days of CPA administration, induced transient lymphocytopaenia, although other blood cell counts were not considerably reduced. Lymphodepletion enabled the transient expansion of CAR‐T cells and subsequent detection in the peripheral blood. No overt acute CAR‐T cell‐related toxicities were observed, although CAR‐T cells showed expansion on day 1 based on the results of the copy number analysis. To the best of our knowledge, this is the first study to prove the feasibility of using a lymphodepleted NHP model for the assessment of acute on‐target/off‐tumor toxicity of human T‐cell therapy products.

The animal model for the toxicity assessment of a newly developed drug should be carefully selected according to the toxicity profiles and endpoints. However, no optimal animal model has been fully established for the toxicity assessment of immune cell‐based products. The immunodeficient murine model is not suitable for the assessment of on‐target/off‐tumor toxicity because of the low degree of homology existing between humans and rodents. Likewise, the investigation of hyperimmune reactions induced by bystander recipient immune cells (i.e. cytokine release syndrome) cannot be performed because of the absence of recipient immune cells in immunodeficient mice. Recently, humanised mouse models, in which human immunity has been developed by transplanting human hematopoietic stem cells, have been established for the investigation of cancer immunotherapeutic agents, such as immune checkpoint inhibitors and cancer vaccines, by grafting human tumors into the humanised mice.^12^, ^13^, ^14^, ^15^ Nevertheless, the lack of cross‐reactivity between humans and rodents continues to hinder the application of this model for the toxicity assessment of CAR‐T cells. The use of NHP models may help in the development of a more informative platform as most of the proteins in NHPs are highly conserved relative to humans. The usefulness of an NHP model has been demonstrated in the safety assessment of immune effector cells, including virally engineered CAR‐T cells.^4^ To facilitate the transient engraftment of allogeneic cellular graft, immunosuppression protocols in NHPs have been well studied, and non‐myeloablative preconditioning is commonly performed to obtain the mixed chimaera and to prevent early graft rejection in allogeneic stem cell transplantation.^16^ Non‐myeloablative conditioning is widely accepted as a safer strategy used for immunosuppression than conventional myeloablative conditioning, and lymphodepletion with FDR and CPA before CAR‐T cell infusion is also considered to increase the persistence of CAR‐T cells and antitumor efficacy.^17^ Considering that experimental myeloablative lymphodepletion, which includes a busulfan‐based regimen^18^, ^19^ or irradiation‐based regimen,^20^ might induce conditioning‐related toxicities that might not be acceptable for the establishment of a toxicologic study model, we followed the most widely accepted non‐myeloablative lymphodepletion protocol in clinical use for CAR‐T cell therapy to deplete the lymphocyte counts in the macaques, and observed successful transient lymphodepletion with minimal and reversible conditioning‐induced toxicities that were consistent with those reported in a previous study.^6^ Moreover, transient expansion of human PBMC‐derived CAR‐T cells was achieved in the CAR‐T group, and no acute on‐target/off‐tumor toxicity was observed when EPHB4 molecules were targeted by their natural ligand, Ephrin B2. These data suggested the safety of utilisation of EPHB4‐CAR‐T cells in terms of acute organ toxicities, although we could not distinguish between the haematological toxicity caused by the conditioning regimen and by the CAR‐T cell product.

As this model is not a tumor‐bearing model, in which the antigen‐expressing tumor has been established, the infused CAR‐T cells might not have been completely activated, which might have resulted in an underestimation of the safety profiles, which indicates one of the limitations of this model. Furthermore, cytokine release syndrome or immune effector cell‐associated neurotoxicity syndrome (ICANS), which could be induced by excess activation of CAR‐T cells by antigen‐expressing tumor cells, could not be accurately reproduced in this model. Another limitation is that immunological reconstitution after completion of immunosuppression may result in the rejection of the cellular graft. Therefore, the lymphodepletion regimen should be further optimised to induce longer lymphopaenia without organ toxicities. Nevertheless, the duration of lymphopaenia was approximately 7 days in our model, which was sufficient for the monitoring of acute on‐target/off‐tumor toxicity. Furthermore, the role of other immune system‐related processes, including antibody‐mediated rejection and innate immunity by natural killer cells or macrophages, in the rejection of xenografts remains unclear.^21^, ^22^, ^23^, ^24^ As the conditioning by FDR/CPA did not affect the numbers of monocytes and neutrophils in this study, these cells could be involved in the impairment of the function of xenogeneic CAR‐T cells. In this context, our NHP model should be carefully utilised for the safety evaluation of CAR‐T cells and should be validated to prove the possible on‐target/off‐tumor toxicity of CAR‐T cells redirected to the shared antigen. Moreover, the lymphodepleted NHP model should be further evaluated by using other CAR‐T cells which are known to exhibit acceptable off‐tumor toxicity (i.e. CD19‐CAR‐T cells) before the validation and application of this model for toxicity assessment. Lastly, even though the NHP model would be useful and considered necessary for the evaluation of on‐target/off‐tumor toxicity, steps should be undertaken to reduce the number of animals used per study or to refine procedures to improve NHP welfare. Notably, we reduced the number of NHPs used for our study and therefore limited the statistical analysis and the generalisation of the results. Development of computational *in silico* cross‐reaction screening technologies would potentially substitute the utilisation of the NHP model for conducting on‐target/off‐tumor study of immune effector cell‐based therapies.

In conclusion, we investigated the on‐target/off‐tumor toxicity of PB‐mediated EPHB4‐CAR‐T cells using a lymphodepleted NHP model. Toxic profiles of immune effector cell products should be thoroughly investigated before applications in the clinic, and animal models should be selected wisely based on the potential immunotoxicity and the limitations of the animal model used for toxicity assessment. Our lymphodepleted NHP model may provide new insights into the safety assessment of immune effector cell‐based therapies as a new investigational intervention.

## Methods

### Transposon plasmid

*piggyBac* transposase plasmid (pCMV‐PB) and *piggyBac* (PB) transposon plasmids used for the stable expression of the EPHB4‐specific CAR transgene (pIRII‐EPHB4‐CAR‐28z) have been described previously,^7^ as was the pIRII‐dEPHB4‐CD80‐CD137L plasmid, which contained an extracellular, transmembrane, 20‐amino acid sequence of the cytoplasmic portion fused to the full‐length CD80 and 4‐1BBL (CD137L) with self‐cleaving 2A sequence, for the production of feeder cells (Supplementary figure 3). For the overexpression of human or cynomolgus *EPHB4*, the full length of human *EPHB4* (NM_004444) or cynomolgus *EPHB4* (XM_005549258) gene fused to puromycin resistance gene and green fluorescent protein (GFP) gene was artificially synthesised (Fasmac Inc., Kanagawa, Japan) and cloned into pIRII transposon plasmids (pIRII‐humanEPHB4‐GFP or pIRII‐cynoEPHB4‐GFP, respectively) (Supplementary figure 3).

### Cell lines and human blood samples

EPHB4‐positive sarcoma cell line RH30 (CRL‐2061) and EPHB4‐negative cell line HEK293 (CRL‐1573) were purchased from the American Type Culture Collection (Manassas, VA, USA). To produce human or cynomolgus EPHB4‐expressing HEK293 cells, we transduced 7.5 μg of the pIRII‐human EPHB4‐GFP or pIRII‐cynoEPHB4‐GFP plasmid into 1 × 10^6^ HEK293 cells along with 7.5 μg of pCMV‐PB for stable expression using the MaxCyte ATX electroporator (MaxCyte, Gaithersburg, MD, USA). The transduced cells were expanded, and GFP‐expressing cells were purified via flow cytometry using the Sony SH800S cell sorter (Sony Biotechnologies Inc., San Jose, CA, USA) to establish human or cynomolgus EPHB4‐expressing HEK293 cells (293‐human EPHB4‐GFP or 293‐cynoEPHB4‐GFP), respectively. HEK293 cells expressing GFP were also generated for the EPHB4‐negative control (293‐GFP). These cells were maintained in the Dulbecco's modified Eagle's medium (Cytiva, Marlborough, MA, USA), supplemented with 10% foetal bovine serum (FBS; Cytiva) and 1% penicillin–streptomycin (Cytiva), and incubated in a 37°C, 5% CO_2_‐containing, humidified incubator.

Blood samples were obtained from normal healthy volunteers, and PBMCs were immediately isolated via density gradient centrifugation using Lymphocyte Separation Medium 1077 (FUJIFILM Wako Pure Chemical Corporation, Osaka, Japan), followed by subjection to multiple washing steps using phosphate‐buffered saline (Nacalai Tesque, Kyoto, Japan) for the generation of CAR‐T or control T cells. The number of live cells was determined using standard trypan blue staining and the Automated Cell Counter model R1 (Olympus, Tokyo, Japan). Reporting has been performed in compliance with the MIATA guidelines.^25^

### Generation of human PBMC‐derived, *piggyBac* transposon‐mediated, EPHB4‐specific CAR‐T cells

The CAR transgene was transduced into fresh, unstimulated PBMCs using the PB transposon system, as per methods described previously^7^, ^26^ (Supplementary figure 4). Briefly, the pCMV‐PB (7.5 μg) and pIRII‐EPHB4‐CAR‐28z (7.5 μg) were introduced into 4 × 10^7^ PBMCs using the MaxCyte ATX electroporator (MaxCyte) with the optimised protocol for the introduction of DNA plasmid into resting T cells (Protocol; RTC 14‐3). Concurrently, pIRII‐dEPHB4‐CD80‐CD137L (15 μg) was introduced into 1 × 10^7^ PBMCs obtained from the same donor using the MaxCyte ATX electroporator to generate the feeder cells. After performing electroporation, CAR‐T cells and the feeder cells were cultured in a complete culture medium (CCM) consisting of ALyS705 Medium (Cell Science & Technology Institute, Inc., Miyagi, Japan), supplemented with 5% artificial serum (Cell Science & Technology Institute, Inc.), IL‐7 (10 ng mL^−1^; Miltenyi Biotec, Bergisch Gladbach, Germany) and IL‐15 (5 ng mL^−1^; Miltenyi Biotec), and incubated in a 37°C, 5% CO_2_‐containing, humidified incubator. Twenty‐four hours after performing electroporation, the feeder cells were UV‐irradiated for inactivation and then co‐cultured with CAR‐T cells in CCM for 14 days in MACS GMP Cell Differentiation Bag‐100 (Miltenyi Biotec). The cells were harvested on day 14 and used for further experiments.

For the expansion of control, non‐transduced T cells, we isolated PBMCs from the same donor from whom the CAR‐T cells had been obtained; the cells were stimulated in a 24‐well plate coated with anti‐CD3 and anti‐CD28 antibodies (Miltenyi Biotec) in CCM for 48 h and then expanded the cells for 12 days.

### Flow cytometry

The expression of EPHB4 was detected via phycoerythrin (PE)‐conjugated EPHB4 antibody (R&D Systems, Inc., Minneapolis, MN, USA) staining. For the detection of EPHB4‐CAR expression, transduced T cells were stained using goat anti‐Ephrin B2 antibody (R&D Systems) and then stained using PE‐conjugated anti‐goat IgG antibody (R&D Systems). Allophycocyanin (APC)‐conjugated anti‐CD3 antibody, APC‐conjugated anti‐CD8a antibody, fluorescein isothiocyanate (FITC)‐conjugated CD4 antibody, FITC‐conjugated anti‐CD45RA antibody, and APC‐conjugated anti‐CCR7 antibody (all from BioLegend) were used for the characterisation of the CAR‐T cell phenotype. To determine the binding capacity of human Ephrin B2 to cynomolgus EPHB4, 293‐human EPHB4‐GFP and 293‐cynoEPHB4‐GFP cells were incubated with the recombinant human Ephrin B2‐Fc Chimera Protein (R&D Systems) for 20 min on ice and then stained using APC‐conjugated anti‐human IgG‐Fc antibody (BioLegend). Detailed antibody information is presented in Supplementary table 2. All flow cytometry data were acquired using BD FACS Accuri C6 Plus (BD Biosciences) and analysed using the FlowJo Software (Tree Star, Inc., Ashland, OR, USA).

### *In vitro* killing assay

To determine the killing effect of the human EPHB4‐CAR‐T cells on 293‐GFP, 293‐human EPHB4‐GFP cells and 293‐cynoEPGB4‐GFP cells, these cells were co‐cultured with EPHB4‐CAR‐T cells for 48 h at an E:T ratio of 1:1. The cell mixture was then collected and mixed with 50000 CountBright™ Absolute Counting Beads (Thermo Fisher Scientific, Inc.) and 7‐amino‐actinomycin D (7AAD). The number of live tumor cells (GFP‐positive, 7AAD‐negative cell fraction) was then determined using flow cytometry until the number of counting beads reached 5000.

### Impedance‐based, tumor cell killing assay

The *in vitro* killing assay was also performed using the xCELLigence^®^ RTCA DP system (ACEA Biosciences, San Diego, CA, USA). Briefly, after background impedance measurement, 1 × 10^4^ RH30 tumor cells were seeded in E‐plates (ACEA Biosciences). Approximately 24 h after seeding, 1 × 10^4^ or 2 × 10^4^ EPHB4‐CAR‐T or control T cells were added for co‐culture. The E‐plates were placed on the RTCA DP Station located inside the incubator (5% CO_2_, 37°C) for continuous recording of impedance. Cell growth and adhesion were monitored every 15 min for approximately 150 h. Electrical impedance was measured and presented as the normalised cell index (CI), and the data were analysed using Software version 2.0 (ACEA Biosciences).

### Animal protocols and monitoring

Cynomolgus macaques were housed at Ina Research Inc., which is fully accredited by the Association for Assessment and Accreditation of Laboratory Animal Care International. Three female and three male cynomolgus macaques, aged 4–5 years, were used in this study. All macaques received 60 mg m^−2^ day^−1^ of FDR for 4 days from day −5 to −2, and 30 mg kg^−1^ day^−1^ of CPA for 2 days on days −4 and −3, intravenously for lymphodepletion^6^ (Figure 1a). To prevent the occurrence of chemotherapy‐induced nausea and vomiting, 2 mg of dexamethasone and 1 mg of granisetron were intravenously administered before the CPA intravenous injection over 10 min. Moreover, 10 mg kg^−1^ of mesna was administered at three time points (immediately, 4 and 8 h after CPA injection) to prevent the development of CPA‐associated haemorrhagic cystitis. A prophylactic antibiotic, cefmetazole sodium, 20 mg kg^−1^ dose^−1^, was provided twice a day during the four days of FDR/CPA injection.

Macaques were divided into the following three groups: No Tx group (one male, one female) that were subjected to only lymphodepletion, NT group (one male, one female) that received control non‐transduced human T cells and CAR‐T group (one male, one female) that received human EPHB4‐CAR‐T cells. On day 0, the T‐cell concentration was adjusted to 3.3 × 10^6^ cells mL^−1^ using normal saline, and 3.3 × 10^6^ kg^−1^ of control T cells or EPHB4‐CAR‐T cells was infused into the NT and CAR‐T groups, respectively. The number of infused cells was determined based on the infusion dose used in ongoing clinical trials targeting sarcomas.^9^, ^10^

All macaques were closely monitored for clinical presentations, including the general condition, food consumption and body weight. For pathological evaluation, all macaques were anaesthetised via intravenous injection of thiopental sodium and were humanely euthanised by exsanguination from the axillary and femoral arteries and veins on day 7 after T‐cell infusion.

### Sample collection, preparation and blood examination

Blood samples were obtained on days −1, 0, 1, 3 and 5 from the femoral vein, and the data for blood count (XN‐2000, Sysmex Inc.), blood chemistry (7180 Clinical Analyzer, Hitachi High‐Tech Technologies) and coagulation tests (CA‐510, Sysmex Inc.) were acquired. Serum samples were stored at −80°C before cytokine analysis and examined for interleukin (IL)‐1β, IL‐2, IL‐6, interferon (IFN)‐γ and tumor necrosis factor (TNF)‐α using Bio‐Plex 200 (Bio‐Rad Laboratories Inc.) and a Non‐Human Primate Cytokine Magnetic Beads panel (EMD Millipore).

### Kinetic analysis for CAR‐T cell persistence

Blood samples were obtained on days 0 (1 and 2 h after T‐cell infusion), 1 and 6 after CAR‐T cell infusion in ethylenediaminetetraacetic acid–sodium (EDTA‐2Na) tubes and stored at −80°C before analysis. Genomic DNA was extracted using the DNeasy Blood and Tissue Kit (Qiagen Inc.). Quantitative polymerase chain reaction (qPCR) for the detection of the specific sequence of the pIRII‐EPHB4‐CAR transgene in 60 ng of genomic DNA from each sample was performed using the Applied Biosystems 7500 Fast Real‐Time PCR System (Thermo Fisher Scientific), TaqMan Fast Universal PCR Master Mix (2×) No AmpErase UNG (Thermo Fisher Scientific Inc.) and the specific primer and probe set (Supplementary table 3). A calibration curve was determined via sequential dilution of pIRII‐EPHB4‐CAR plasmid samples (10^8^–10^0^ copies/well), and the copy number of the EPHB4‐CAR transgene was calculated as copies/60 ng of genomic DNA from each sample.

### Pathological analysis

On day 7 after CAR‐T cell infusion, organs, including the heart, spleen, liver, gallbladder, kidney, brain and thoracic spinal cord, were harvested from each macaque; organ specimens were fixed with 10% formaldehyde and stained using haematoxylin and eosin. All pathological specimens were carefully examined by expert pathologists.

## Conflict of interest

Hidemi Mochizuki, Kengo Sakamoto and Akihito Shimoi are employees of Ina Research, Inc. The authors have no other conflicts of interest.

## Author contributions

**Shigeki Yagyu:** Conceptualization; Data curation; Formal analysis; Funding acquisition; Investigation; Methodology; Resources; Supervision; Writing‐original draft; Writing‐review & editing. **Hidemi Mochizuki:** Data curation; Formal analysis; Investigation; Resources; Writing‐original draft; Writing‐review & editing. **Kumiko** **Yamashima:** Data curation; Investigation; Writing‐review & editing. **Hiroshi Kubo:** Data curation; Investigation; Writing‐review & editing. **Shoji Saito:** Conceptualization; Investigation; Writing‐review & editing. **Miyuki Tanaka:** Conceptualization; Investigation; Writing‐review & editing. **Kengo Sakamoto:** Data curation; Formal analysis; Investigation; Methodology; Resources; Writing‐review & editing. **Akihito** **Shimoi:** Conceptualization; Data curation; Formal analysis; Investigation; Methodology; Resources; Writing‐review & editing. **Yozo Nakazawa:** Conceptualization; Investigation; Methodology; Resources; Supervision; Writing‐review & editing.
