# Supplementary material for: A lymphodepleted non‐human primate model for the assessment of acute on‐target and off‐tumor toxicity of human chimeric antigen receptor‐T cells
Source: Clin Transl Immunology. 2021 Jun 3;10(6):e1291. doi: 10.1002/cti2.1291 (PMC8175993; doi:10.1002/cti2.1291)
Supplement: Supplementary file 1 [file CTI2-10-e1291-s001.pdf]

Supplementary figure 1

Corresponding Author; Shigeki Yagyu

```
MACACA MELRALLCWASLAAALEETLLNTKLETADLKWVTFPQVDGQWEELSGLDEEQHSVRTYEV   60
_HUMAN MELRVLLCWASLAAALEETLLNTKLETADLKWVTFPQVDGQWEELSGLDEEQHSVRTYEV
MACACA CDVQRAPGQAHWLRTGWVPRRGAVHVYATLRFTMLECLSLPRAGRSCKETFTVFYYESDA   120
_HUMAN CDVQRAPGQAHWLRTGWVPRRGAVHVYATLRFTMLECLSLPRAGRSCKETFTVFYYESDA
MACACA DTATALTPAWMENPYIKVDTVAAEHLTRKRPGAEATGKVNVKTLRLGPLSKAGFYLAFQD   180
_HUMAN DTATALTPAWMENPYIKVDTVAAEHLTRKRPGAEATGKVNVKTLRLGPLSKAGFYLAFQD
MACACA QGACMALLSLHLFYKKCAQLTVNLTRFPETVPRELVVPVAGSCVVDAVPAPGPSPSLYCR   240
_HUMAN QGACMALLSLHLFYKKCAQLTVNLTRFPETVPRELVVPVAGSCVVDAVPAPGPSPSLYCR
MACACA EDGQWAEQPVTGCSCAPGFEAAEGNTKCRACAQGTFKPLSGEGSCQPCPANSHSNNIGSA   300
_HUMAN EDGQWAEQPVTGCSCAPGFEAAEGNTKCRACAQGTFKPLSGEGSCQPCPANSHSNTIGSA
MACACA VCQCRIGYFRARTDPRGAPCTTPPSAPRSVVSRLNGSSLHLEWSAPLESGGREDLTYALR   360
_HUMAN VCQCRVGYFRARTDPRGAPCTTPPSAPRSVVSRLNGSSLHLEWSAPLESGGREDLTYALR
MACACA CRECRPGGSCAPCGGDLTFDPGPRDLVEPWVVVRGLRPDFTYTFEVTALNGVSSLATGPV   420
_HUMAN CRECRPGGSCAPCGGDLTFDPGPRDLVEPWVVVRGLRPDFTYTFEVTALNGVSSLATGPV
MACACA PFEPVNVTTDREVPPAVSDIRVTRSSPSSLSLAWAVPRAPSGAVLDYEVKYHEKGAEGPS   480
_HUMAN PFEPVNVTTDREVPPAVSDIRVTRSSPSSLSLAWAVPRAPSGAVLDYEVKYHEKGAEGPS
MACACA SVRFLKTSENRAELRGLKRGASYLVQVRARSEAGYGPFGQEHHSQTQLDENEGWREQLAL   540
_HUMAN SVRFLKTSENRAELRGLKRGASYLVQVRARSEAGYGPFGQEHHSQTQLDESEGWREQLAL
MACACA IAGTAVVGVVLVLVVIVVAVLCLRKQSNGREAEYSDKHGQYLIGHGTKVYIDPFTYEDPN   600
_HUMAN IAGTAVVGVVLVLVVIVVAVLCLRKQSNGREAEYSDKHGQYLIGHGTKVYIDPFTYEDPN
MACACA EAVREFAKEIDVSYVKIEEVIGAGEFGEVCRGRLKAPGKKESCVAIKTLKGGYTERQRRE   660
_HUMAN EAVREFAKEIDVSYVKIEEVIGAGEFGEVCRGRLKAPGKKESCVAIKTLKGGYTERQRRE
MACACA FLSEASIMGQFEHPNIIRLEGVVTNSMPVMILTEFMENGALDSFLRLNDGQFTVIQLVGM   720
_HUMAN FLSEASIMGQFEHPNIIRLEGVVTNSMPVMILTEFMENGALDSFLRLNDGQFTVIQLVGM
MACACA LRGIASGMRYLAEMSYVHRDLAARNILVNSNLVCKVSDFGLSRFLEENSSDPTYTSSLGG   780
_HUMAN LRGIASGMRYLAEMSYVHRDLAARNILVNSNLVCKVSDFGLSRFLEENSSDPTYTSSLGG
MACACA KIPIRWTAPEAIAFRKFTSASDAWSYGIVMWEVMSFGERPYWDMSNQDVINAIEQDYRLP   840
_HUMAN KIPIRWTAPEAIAFRKFTSASDAWSYGIVMWEVMSFGERPYWDMSNQDVINAIEQDYRLP
MACACA PPPDCPTSLHQLMLDCWQKDRNARPRFPQVVSALDKMIRNPASLKIVARENGGDLLRIGV   900
_HUMAN PPPDCPTSLHQLMLDCWQKDRNARPRFPQVVSALDKMIRNPASLKIVARENGGDLLRIGV
MACACA TLAGHQKKILASVQHMKSQAKPGAPGGTGGPTPQY   935
_HUMAN TLAGHQKKILASVQHMKSQAKPGTPGGTGGPAPQY   935
```

Supplementary figure 1

Homology of the extracellular domain of human and cynomolgus EPHB4. Extracellular portion of EPHB4 amino acid sequence was highlighted in green (cynomolgus macaque; A0A2K5V953) and red (Homo Sapiens; P54760). Homology of the extracellular portion of human and cynomolgus EPHB4 was >99%.

Supplementary figure 2

Corresponding Author; Shigeki Yagyu

No Tx group (Male)

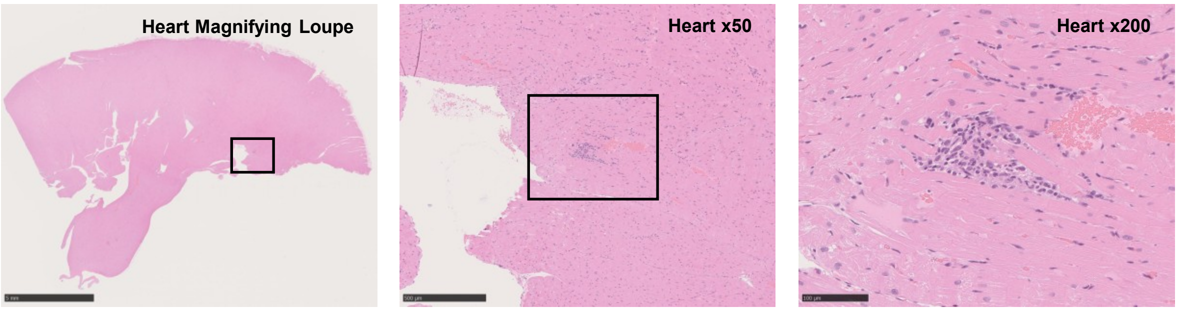

Supplementary figure 2
Histological image of heart in PBMC group. The infiltration of mononuclear cells was detected.

Supplementary figure 3

Corresponding Author; Shigeki Yagyu

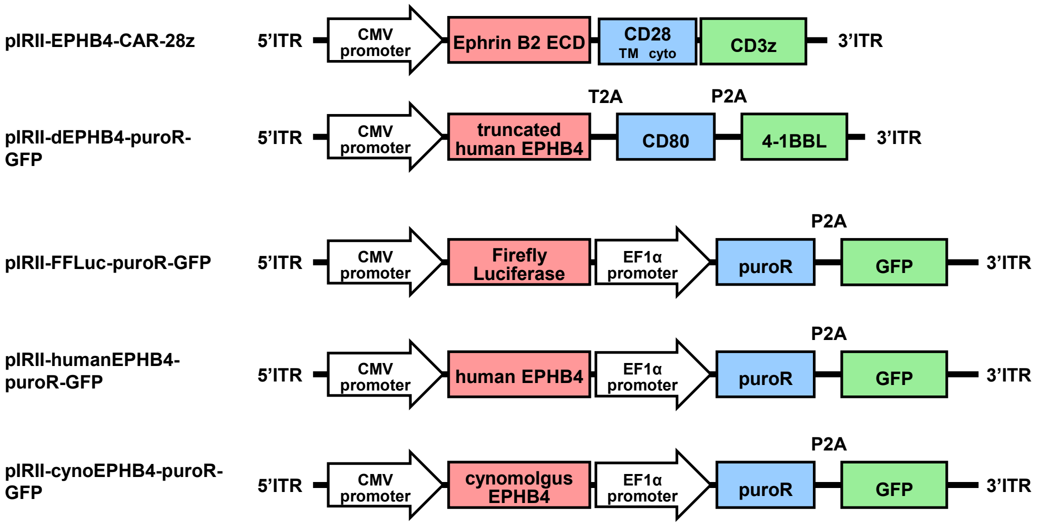

Supplementary figure 3
Transposon plasmids. ECD; Extra cellular domain, ITR; Inverted Tandem Repeat, puroR; puromycin resistant gene, GFP; Green fluorescent protein gene.

Supplementary figure 4

Corresponding Author; Shigeki Yagyu

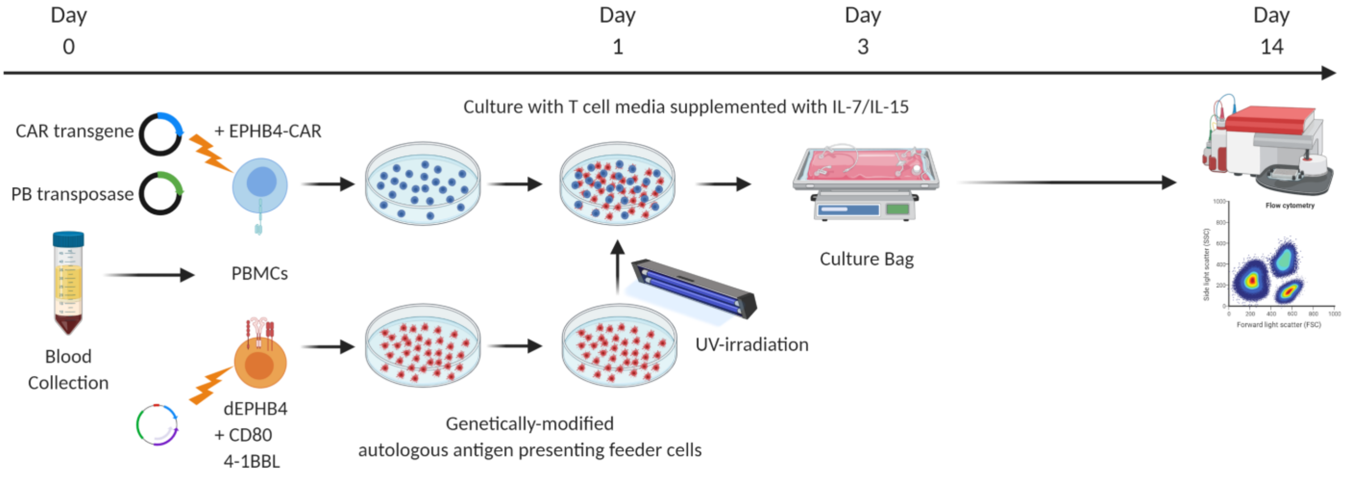

Supplementary figure 4
PB-EPHB4-CAR-T cell manufacturing scheme. Detailed manufacturing procedure was described in Materials and Methods.

Supplementary Table 1

Corresponding Author; Shigeki Yagyu

Hematology

Parameters	(Unit)	n	Min	Max
HGB	(g/dL)	525	10.8	15.9
HCT	(%)	525	35.1	51.1
Retic	(10^9/L)	525	11.9	115.4
WBC	(10^3/μL)	524	3.48	22.08
Neut	(10^3/μL)	518	0.47	11.27
Mono	(10^3/μL)	522	0.14	1.11
Lymph	(10^3/μL)	521	2.24	15.26
PLT	(10^3/μL)	525	157	595

Biochemistry

Parameters	(Unit)	n	Min	Max
AST	(U/L)	901	13	81
ALT	(U/L)	902	8	122
LDH	(U/L)	780	141	581
CK	(U/L)	838	58	1316
CRP	(mg/dL)	93	0.01	0.64
BIL	(mg/dL)	905	0.03	0.31
TP	(g/dL)	505	6.30	8.82
ALB	(g/dL)	502	3.31	4.95
UN	(mg/dL)	913	10.1	32.3
CRE	(mg/dL)	911	0.47	1.01
Na	(mEq/L)	913	143.4	166.9
K	(mEq/L)	914	3.04	6.19

Supplementary Table 1
In-house data of blood examination. Two to four-years-old healthy cynomolgus macaques housed in Ina Research Inc. from 2016 to 2019 were subjected to a blood examination to obtain the reference values. The minimum and maximum values of each parameter were determined.

Supplementary Table 2

Corresponding Author; Shigeki Yagyu

Name	clone	Product#	Company
Goat anti Ephrin B2	polyclonal	AF496	R&D SYSTEMS
PE anti-goat IgG Fc	polyclonal	F0107	R&D SYSTEMS
APC anti CD3	UCHT1	300412	Biolegend
APC anti-CD8a	HIT8a	300912	Biolegend
FITC anti-CD4	OKT4	317408	Biolegend
FITC anti-CD45RA	HI100	304106	Biolegend
APC anti-CCR7	G043H7	353214	Biolegend
Recombinant human Ephrin B2-Fc chimera protein	-	7397-EB	R&D SYSTEMS
APC anti human IgG Fc	HP6017	409306	Biolegend

Supplementary Table 2
List of antibodies used in this study.

Corresponding Author; Shigeki Yagyu

(Forward Primer) 5'-GACTACATGAACATGACTCCCCG-3'
(Reverse Primer) 5'-GTCCAAAACATCGTACTCCTCTCT-3'
(probe) 5' FAM-ACCCGCAAGCATTACCAGCCCT-TAMRA 3'

Supplementary Table 3
Primer and Probe set for the detection of EPHB4-CAR transgene.
